# Consumption habits of school canteen and non-canteen users among Norwegian young adolescents: a mixed method analysis

**DOI:** 10.1186/s12887-018-1299-0

**Published:** 2018-10-16

**Authors:** Arthur Chortatos, Laura Terragni, Sigrun Henjum, Marianne Gjertsen, Liv Elin Torheim, Mekdes K Gebremariam

**Affiliations:** 1 Department of Nursing and Health Promotion, Faculty of Health Sciences, OsloMet – Oslo Metropolitan University, P.O. Box 4, St Olavs plass, 0130 Oslo, Norway; 2 Department of Epidemiology, Fielding School of Public Health, University of California, Los Angeles (UCLA), Los Angeles, CA USA; 3 Department of Nutrition, Institute of Basic Medical Sciences, University of Oslo, P.O. Box 1046 Blindern, N-0317 Oslo, Norway

**Keywords:** Dietary behaviours, School lunch, Adolescents

## Abstract

**Background:**

Food/drinks available to adolescents in schools can influence their dietary behaviours, which once established in adolescence, tend to remain over time. Food outlets’ influence near schools, known to provide access to unhealthy food/drinks, may also have lasting effects on consumption behaviours. This study aimed to gain a better understanding of the consumption habits of adolescents in the school arena by comparing different personal characteristics and purchasing behaviours of infrequent and regular school canteen users to those never or seldom using the canteen.

**Methods:**

A convergent mixed methods design collected qualitative and quantitative data in parallel. A cross-sectional quantitative study including 742 adolescents was conducted, with data collected at schools via an online questionnaire. Focus group interviews with students and interviews with school administrators formed the qualitative data content. Quantitative data were analysed using descriptive statistics and logistic regression; thematic content analysis was used to analyse qualitative data.

**Results:**

Sixty-seven percent of adolescents reported never/rarely using the school canteen (NEV), whereas 13% used it ≥2 times per week (OFT). When the two groups were compared, we found a significantly higher proportion of the NEV group were female, having parents with a high education, and with a high self-efficacy, whilst a significantly higher proportion of the OFT group consumed salty snacks, baked sweets, and soft-drinks ≥3 times per week, and breakfast at home < 5 days in the school week. The OFT group had significantly higher odds of purchasing food/drink from shops near school during school breaks and before/after school compared to the NEV group (adjusted odds ratio (aOR) = 1.80, 95% CI 1.07–3.01, and aOR = 3.61, 95% CI 2.17–6.01, respectively). The interviews revealed most students ate a home packed lunch, with the remainder purchasing either at the school canteen or at local shops.

**Conclusions:**

Students using the canteen often are frequently purchasing snacks and sugar-soft drinks from shops near school, most likely owing to availability of pocket money and an emerging independence. School authorities must focus upon satisfying canteen users by providing desirable, healthy, and affordable items in order to compete with the appeal of local shops.

**Electronic supplementary material:**

The online version of this article (10.1186/s12887-018-1299-0) contains supplementary material, which is available to authorized users.

## Background

The school environment is an arena where many dietary norms and habits are established which potentially affect the individual throughout their future lives [1]. Owing to the considerable amount of time adolescents spend at school during the average weekday, it has been estimated that approximately one third of their food and drink is consumed in the school environment [2, 3].

Environments which encourage a high energy intake and sedentary behaviour amongst adolescents are termed obesogenic environments, and such environments are considered to be one of the main elements behind the rapid increase in overweight and obesity among children and adolescents [4].

In this regard, the local food environment of schools, including arenas such as supermarkets and convenience stores close to the schools, is an environmental influence potentially affecting the quality of the food intake of attending adolescents [5]. Providing healthy food and drinks to adolescents in schools via canteens or vending machines plays an important role in modelling a healthy diet, particularly for those who may not have access to healthy food outside school hours, thereby making school nutrition policies a powerful tool for improving students’ nutritional status and academic achievement [6]. Yet in the school environment, foods consumed are not always obtained from on-campus sources. Research upon supermarkets and convenience stores located in the vicinity of schools has reported that these venues provide an increased accessibility to unhealthy foods and drink for school-going adolescents [7].

The Øvre Romerike region, located in the eastern part of Norway, has a total area of 2,055,550 km^2^, and composed of 6 municipalities housing approximately 100,000 people [8]. The 2016 average net income for all households in the region was 456,667 NOK, compared to the national average of 498,000 NOK for the same period [9]. In our recent investigation upon adolescents in Øvre Romerike, we reported that 33% of participants purchased food or drink in their school canteen at least once a week [10]. In addition, 27% and 34% of participants reported purchasing food and drinks from shops around schools one or more times a week, either during school breaks or on their way to or from school, respectively [10].

Investigations on adolescent behaviour in Norway and elsewhere have reported similar results, whereby approximately 30% of school-going adolescents visit local food stores for nourishment, whilst the majority are consuming their lunches at school [11, 12].

In Norway, the average school day includes a lunch period in the middle of the day [13], and most students travel to school with a home packed lunch, usually consisting of bread slices with various toppings [14, 15]. School canteens are often run by catering staff, with students in need of more practical education sometimes included in food preparation and selling. It is not uncommon for the canteen to be managed on a daily or occasional basis by students together with a teacher as a part of their education. School canteens most commonly offer baguettes, waffles, milk (regular or chocolate), juice, cakes and, perhaps, fruit [16, 17]. The Norwegian Directorate of Health regularly publishes guidelines concerning school meals and eating environments, with the most recent published in 2015 [18]. The latest guidelines offer suggestions regarding topics such as length of meal times, hygiene, fresh water accessibility, the absence of sugar-rich foods and drinks, and the reduction of saturated fats on offer. The guidelines are published as a tool to assist school administration in their management of school canteens.

Eating behaviour amongst adolescents is a complex theme often involving an interplay of multiple influences and factors such as peer influence [19] and a desire to socialise whilst eating [20], a combination which often leans toward unhealthy eating practices. Furthermore, it is not uncommon for young Norwegian teens to receive pocket money [21], and this emerging autonomy aided by pocket money increases the prospect for a disruption of dietary behaviour established in the home [22].

As the school food environment has such a significant impact on food choices [23, 24], a better understanding of adolescent’s consumption behaviour demands further attention. In particular, understanding student’s shift away from home packed lunches and canteen foods towards the appeal of off-campus shop food is necessary for implementing the successful promotion of healthier lunch alternatives at school.

The aim of the present study was to gain a better understanding of the consumption habits of adolescents in the Norwegian school lunch arena. Unlike previous ESSENS studies, here we use quantitative data combined with qualitative interviews among adolescents and school administration, in order to explore the purchasing behaviour and lifestyle demographics of the sample grouped as frequent and infrequent school canteen users compared to those never or rarely using the canteen.

## Methods

### Design and sample

The participants in this study were students and staff from eleven secondary schools participating in the Environmental determinantS of dietary behaviorS among adolescENtS (ESSENS) cross-sectional study [10, 25]. Recruitment of students and staff was initiated by our making contact with principals of the twelve secondary schools in the Øvre Romerike district, after first having received permission from district school leaders. The school principals were each sent a letter detailing key elements of the proposed intervention, as well as information regarding the ESSENS study, together with a permission form requesting their school’s participation. Of the twelve secondary schools invited to participate in the study, eleven accepted the invitation.

In this mixed method approach, our sample were grouped as being part of either a quantitative or qualitative data source.

### Recruitment of sample

#### Quantitative recruitment

In October 2015 we recruited 8th grade adolescents for participation in a questionnaire survey. An informative letter was sent home with all 1163 adolescents in the 8th grade (average age of 12–13 years) from the 11 participating schools, containing a consent form for signing and with additional questions relating to parental education levels. A total of 781 (67%) received parental consent for participation. As the range of ages of the sample represents the lower end of the adolescent scale (10–19 years), the use of the term ‘adolescent’ here implies ‘young adolescent’. A total of 742 adolescents (64% of those invited and 95% of those with parental consent) participated in the survey. Quantitative data collection took place between October and December 2015.

#### Qualitative recruitment

Recruitment of adolescents to participate in the qualitative part of the study was also facilitated by approaching principals of district schools as described above, and was completed between October 2015 and January 2016. Six of the 11 participating schools were selected for qualitative data collection based upon criteria such as location (being in one of the six municipalities of Øvre Romerike), and size (based upon number of students attending). The aim was to include schools with a varied profile, with proximity to city centers, shops, and collective transport as determining factors. Thereafter a selection process for participation in the focus groups was conducted, whereby two students per class were sought after, representing both sexes. Further inclusion criteria stipulated that the students be in the 9th grade, had attended Food and Health classes, and currently lived in the Øvre Romerike area with either one or both parents.

### Data collection

#### Quantitative data

A web-based questionnaire was used to collect data from the adolescents, using the LimeSurvey data collection tool. The questionnaires were answered at school, taking approximately 30–45 min to complete, and queried respondents about their nutritional intake, parental rules regarding food and drink consumption, students’ school canteen and surrounding shop use, physical activity, and sedentary behaviour habits. Research group members were present during data collection to answer questions and make sure the adolescents responded independently from each other. The questionnaire relating to food behaviours completed by the sample is available online (see Additional file 1: Appendix 1 ESSENS questionnaire relating to food behaviours).

A pilot test of the survey was conducted parallel with this process in a neighboring municipality with similar age students from the 8th grade (*n* = 23). The students spent approximately 30–40 min to complete the survey, and then provided feedback regarding comprehension. The questionnaire was subsequently shortened and some questions rephrased for clarity. The results of the pilot test were not included in the final results.

#### Qualitative data

Focus group interviews were conducted over a period of 10 weeks, from November 2015 to January 2016. Focus group settings were favoured as they provide a more relaxed setting for data collection, facilitating the flow of a natural conversation amongst peers, especially when adult researchers interact with young subjects [26].

Six focus group interviews including a total of 55 students (29 girls, 26 boys) from the 9th grade with an average age of 13–14 years were conducted. Interviews had a duration of approximately 60 min. In addition, interview sessions with headmasters and teachers for the 9th grade students from the participating schools were also conducted. Interviews with 6 teachers (4 women and 2 men) and 6 headmasters (3 women and 3 men) were conducted from October 2015 to January 2016. The interviews with principals and teachers were each conducted separately.

Qualitative data collection took place at the selected schools using an audio recorder, with a semi-structured interview guide used for the interviews, partially inspired by a previous study conducted amongst 11–13 year old Norwegian adolescents [27]. The main themes explored by the focus group sessions were students’ eating habits, their definition of healthy and unhealthy food, attitudes towards and their impact upon diet and physical activity, as well as the student’s assessment of opportunities and barriers attached to health-promoting behaviour. School administration interviews probed food availability and meals served at the school, as well as physical activity options available for students at the schools. The interview guides used for the focus groups and the school administration are available online (see Additional file 2: Appendix 2 Interview guide for focus group interviews, and Additional file 3: Appendix 3 Interview guide for headmasters and teachers).

Interviews were transcribed verbatim, with names of the participants and of the schools anonymised. Interviews were analysed using a thematic analysis approach [28]. Codes were developed after an initial reading of all the transcripts and were based on the main interview questions, prior research, and emergent concepts from the current data. The initial codes were discussed among researchers and a codebook was developed. The codes were further refined during coding of subsequent transcripts. Codes were then successively grouped into general themes. The data analysis was supported by the use of NVivo software (version 10.0; QSR International, Cambridge, Mass).

Pilot testing of the intended focus group question guide was performed in October 2015 in a school belonging to a neighbouring district. After written consent was obtained from the principal of the school, 6 students from the 9th grade were selected by a 9th grade teacher from the school. Three girls and 3 boys were included in the focus group pilot test. A moderator conducted the focus group following an interview guide in order to test comprehension and flow of the planned themes. The pilot test proved effective and consequently no changes were made to the interview guide. Data from the pilot testing was not included in the results of the study.

Recruitment of school staff for participation in in-depth interviews was also facilitated by the agreement with administrative school leaders as described above. A written invitation was sent to principals and teachers of the 9th grade classes from the same 6 schools participating in focus group interviews. Those agreeing were later contacted by phone to arrange a place and time for the interview.

Pilot testing of school staff interviews was performed in October 2015 in a school belonging to a neighbouring district. Two interviews were conducted with one headmaster and one teacher separately in order to assess the comprehension and flow of the various themes probed, as well as the time used for the interview. Data from the pilot testing was not included in the results of the study.

### Measures

The following measures obtained from the questionnaire were used in the quantitative analyses of the present study.

#### Sociodemographic measures

Two questions assessing parental education (guardian 1 and guardian 2) were included on the parental informed consent form for the adolescent. Parental education was categorised as low (12 years or less of education, which corresponded to secondary education or lower) or high (13 years or more of education, which corresponded to university or college attendance). The parent with longest education, or else the one available, was used in analysis. Participants were divided into either ethnic Norwegian or ethnic minority, with minorities defined as those having both parents born in a country other than Norway [29].

#### Dietary behaviours

Frequency of carbonated sugar-sweetened soft-drink intake (hereafter referred to as soft-drinks) during weekdays was assessed using a frequency question with categories ranging from never/seldom to every weekday. Weekday frequency was categorised as less than three times per week and three or more times per week.

The questions assessing the intake of soft-drinks have been validated among 9- and 13-year-old Norwegians using a 4-day pre-coded food diary as the reference method, and moderate Spearman’s correlation coefficients were obtained [30].

Consumption of fruits and vegetables (raw and cooked) were assessed using frequency questions with eight response categories ranging from never/seldom to three times per day or more. These were further categorised as less than five times per week and five or more times per week. The questions assessing intake of fruits and vegetables have been validated among 11-year-olds with a 7-day food record as the reference method and were found to have a satisfactory ability to rank subjects according to their intake of fruits and vegetables [31].

The consumption of snacks [sweet snacks (chocolate/sweets), salty snacks (e.g. potato chips), and baked sweets (sweet biscuits/muffins and similar)] was assessed using three questions with seven response categories ranging from never/seldom to two times per day or more. These were further categorised as less than three times per week and three or more times per week. Acceptable to moderate test-retest reliability have been obtained for these measures of dietary behaviours in a previous Norwegian study conducted among 11-year-olds [27].

Self-efficacy related to the consumption of healthy foods was assessed using a scale with six items [e.g. Whenever I have a choice of the food I eat. .., I find it difficult to choose low-fat foods (e.g. fruit or skimmed milk rather than ‘full cream milk’)]. Responses were further categorised as those with ‘high’ self-efficacy (score of 3.5 or higher, from a scale of 1–5) or ‘low’ self-efficacy (under 3.5, from a scale of 1–5). The scale has been found to have adequate reliability and factorial validity among 13-year-olds [32].

Adolescents’ breakfast consumption was assessed using one question asking the adolescents on how many schooldays per week they normally ate breakfast. The answers were categorised as those eating breakfast 5 times per week or less than 5 times per week. This question has shown evidence of moderate test-retest reliability (percentage agreement of 83 and 81% respectively for weekday and weekend measures) and moderate construct validity (percentage agreement of 80 and 87% respectively for weekday and weekend measures) among 10–12 year old European children [27].

#### Food/drink purchases in school environment

The adolescents were asked how often they purchased foods or drinks from school canteens and on their way to and from school (answer categories ranging from ‘never’ to ‘every day’). The frequency of purchase of food/drinks at the school canteen were then re-categorised into ‘never/rarely’, ‘once per week’, or ‘two or more times per week’. The frequency of purchase of food/drinks at off-campus food stores were re-categorised into ‘never/rarely’, or ‘one or more times per week’. They were also asked about the presence of food sales outlets (e.g. supermarket, kiosk, or gas station) in a walking distance from their school (with answer categories ‘none’, ‘yes, one’, ‘yes, two’, and ‘yes, more than two’), with results categorised as ‘less than 3’ or ‘3 or more’.

Further details regarding data collection and methodology in the ESSENS study have been described previously [10]. Ethical clearance for the study was obtained from the Norwegian Social Science Data Service (NSD 2015/44365). Written informed consent was obtained from all parents of participating students.

### Statistical analyses

The study sample was divided into three groups, those who reported ‘never or rarely’ using the school canteen (NEV), those using the canteen once per week (SEL), and those reporting use of the school canteen ‘two or more times during the week’ (OFT). Results are presented as frequencies (%), with chi-square tests performed to examine differences in sociodemographic, behavioural, and dietary characteristics between the three groups. A further logistic regression analysis was performed to assess the adjusted associations between canteen use and dietary habits (salty snacks, baked sweets, soft-drinks, and home breakfast frequency). Adjustment was made for significant sociodemographic and behavioural characteristics (gender, parental education, self-efficacy) and shop use (during school break and before/after school). Logistic regression was also used to explore the adjusted association between visiting shops during school breaks or before/after school (‘never/rarely’, ‘one or more times per week’), and use of canteen (NEV, SEL, OFT). Results are presented as crude odds ratios (cOR) and adjusted odds ratios (aOR) with 95% confidence intervals (95% CIs). Cases with missing data were excluded from relevant analyses. Because schools were the unit of measurement in this study, we checked for clustering effect through the linear mixed model procedure. Only 3% of the unexplained variance in the dietary behaviours investigated was at the school level, hence adjustment for clustering effect was not done.

A significance level of 0.05 was used. All analyses were performed using SPSS 24.0 (IBM Corp, Armonk, NY, USA).

## Results

### Sample demographics

The mean age of the survey sample was 13.6 years ±0.3 standard deviation, 53% of participants were females, and 60% had parents with a high level of education (≥13y, Table 1). The proportion of adolescents who never or rarely used the school canteen was 67.4%. When comparing demographics and behavioural characteristics for the sample grouped as those using the school canteen never/rarely (NEV), those using the canteen once a week (SEL, 19.7%), and those using the canteen two or more times a week (OFT, 12.9%), we found a significantly higher proportion of the NEV group were female, having parents with a high education, and with a high self-efficacy.

**Table 1:** Sociodemographic and behavioural characteristics of sample total (*n* = 742)^a^, and grouped by frequency of canteen use

Demographics	Total	NEV^b^	SEL	OFT	*P* value^c^
	*n* (%)	*n* (%)	*n* (%)	*n* (%)	
Gender (female), *n* = 720	386 (53.6)	277 (57.1)	66 (46.5)	43 (46.2)	0.03
Parental education (≥13y), *n* = 690	417 (60.4)	306 (65.5)	71 (52.6)	40 (45.5)	< 0.001
Ethnicity (minority), n = 720	64 (8.9)	44 (9.1)	7 (4.9)	13 (14.0)	0.06
Self-efficacy (high), *n* = 684	366 (53.0)	271 (58.2)	55 (41.0)	40 (47.6)	0.001

^a^Discrepancies in size from sample total may exist owing to missing values

^b^NEV: adolescents never or rarely using the school canteen (*n* = 485); SEL: adolescents using the school canteen once a week (*n* = 142); OFT: adolescents using the school canteen ≥2 times a week (*n* = 93)

^c^Chi-square test between frequency of canteen use groups

### Canteen use and dietary habits

When analysing the dietary habits for the sample grouped by frequency of canteen use, a significantly higher proportion of the OFT group reported consuming salty snacks, baked sweets, and soft-drinks ≥3 times per school week, and a significantly higher proportion of the NEV group reported eating breakfast 5 days in the school week compared to the SEL and OFT groups (Table 2). A multiple logistic regression was conducted to assess whether these significant associations between canteen use and dietary behaviours persisted after adjustment for gender, parental education, self-efficacy, and use of shops (both during and before/after school). The difference between NEV, SEL, and OFT adolescents regarding baked sweets thereafter became non-significant. However, the difference between NEV and OFT adolescents regarding salty snacks, soft-drinks, and breakfast consumption remained significant, indicating that adolescents using the canteen ≥2 times per week had increased odds for consuming salty snacks and soft-drinks (aOR 2.05, 95% CI 1.07–3.94, *p* < 0.03, and aOR 2.32, 95% CI 1.16–4.65, *p* < 0.02, respectively, data not shown). Additionally, the OFT group had reduced odds of consuming breakfast at home daily (aOR 0.48, 95% CI 0.28–0.80, *p* = 0.005, data not shown). No significant differences between the three groups were found for the other food items explored.

**Table 2:** Frequency of food consumption for sample grouped by frequency of canteen use (*n* = 742)^a^

Dietary habits		NEV^b^	SEL	OFT	*P* value^c^
	*n*	*n (%)*	*n (%)*	*n (%)*	
Fruit					
< 5 times/week	346	225 (46.4)	72 (51.5)	49 (52.7)	
≥ 5 times/week	373	260 (53.6)	69 (48.9)	44 (47.3)	0.40
Vegetables (raw, incl. salad)					
< 5 times/week	410	264 (54.9)	87 (61.7)	59 (63.4)	
≥ 5 times/week	305	217 (45.1)	54 (38.3)	34 (36.6)	0.16
Vegetables (cooked, not incl. potatoes)					
< 5 times/week	461	301 (62.3)	96 (68.6)	64 (68.8)	
≥ 5 times/week	255	182 (37.7)	44 (31.4)	29 (31.2)	0.25
Chocolate/sweets					
< 3 times/week	549	378 (77.9)	106 (75.2)	65 (70.7)	
≥ 3 times/week	169	107 (22.1)	35 (24.8)	27 (29.3)	0.30
Salty snacks					
< 3 times/week	619	424 (88.5)	123 (87.9)	72 (78.3)	
3 times/week	92	55 (11.5)	17 (12.1)	20 (21.7)	< 0.03
Baked sweets					
< 3 times/week	641	440 (90.7)	127 (92.0)	74 (80.4)	
≥ 3 times/week	74	45 (9.3)	11 (8.0)	18 (19.6)	0.007
Soft-drinks^d^					
< 3 times/week	648	446 (92.1)	128 (91.4)	74 (80.4)	
≥ 3 times/week	68	38 (7.9)	12 (8.6)	18 (19.6)	0.002
Eat breakfast home^d^					
< 5 times/week	227	136 (28.0)	49 (34.5)	42 (45.2)	
5 times/week	493	349 (72.0)	93 (65.5)	51 (54.8)	0.003

^a^Discrepancies in size from sample total may exist owing to missing values

^b^NEV: adolescents never or rarely using the school canteen (*n* = 485); SEL: adolescents using the school canteen once a week (*n* = 142); OFT: adolescents using the school canteen ≥2 times a week (*n* = 93)

^c^Chi-square test between frequency of canteen use groups

^d^Evaluated on the Monday-Friday school week

### School environment

When comparing the frequency of food purchases at shops during school breaks or on the way to/from school for the NEV, SEL, and OFT groups, we found that a significantly higher proportion of OFT adolescents reported purchasing food/drink from a shop near school either during school breaks or else before or after school, one or more times during the week (Table 3). Logistic regression analyses revealed that the OFT group had significantly higher odds of purchasing food/drink from a shop near school, either during school breaks or else before or after school, than the NEV group (aOR = 1.80, 95% CI 1.07–3.01, and aOR = 3.61, 95% CI 2.17–6.01, respectively, Table 4).

**Table 3:** Food/drink purchases from shops and shop numbers encountered for sample grouped for canteen use (*n* = 742)^a^

Purchase habits		NEV^b^	SEL	OFT	P value
	*n*	*n (%)*	*n (%)*	*n (%)*	
Purchase food/drink from shop near school during school break					
Never/rarely	524	358 (74.0)	109 (77.3)	57 (62.6)	
≥1 time/week	192	126 (26.0)	32 (22.7)	34 (37.4)	< 0.05
Purchase food/drink from shop near school before/after school					
Never/rarely	477	347 (72.1)	89 (62.7)	41 (44.1)	
≥1 time/week	239	134 (27.9)	53 (37.3)	52 (55.9)	< 0.001
Number of shops within walking distance from school					
< 3 shops	359	253 (52.2)	69 (49.6)	37 (39.8)	
≥3 shops	358	232 (47.8)	70 (50.4)	56 (60.2)	0.09

^a^Discrepancies in size from sample total may exist owing to missing values

^b^NEV: adolescents never or rarely using the school canteen (*n* = 485); SEL: adolescents using the school canteen once a week (*n* = 142); OFT: adolescents using the school canteen ≥2 times a week (*n* = 93)

**Table 4:** Odds ratios for the association between visiting local shops (*n* = 651) and use of school canteen

			Crude		Adjusted^a^	
Outcome	Group	*n (%)*	cOR^b^ (95% CI)	*P* value	aOR (95% CI)	*P* value
Visit shop during school break ≥1 time week	NEV^c^	447 (68.7)	1.00	0.02	1.00	0.05
	SEL	126 (19.4)	0.95 (0.60–1.50)		0.89 (0.55–1.43)	
	OFT	78 (12.0)	2.00 (1.21–3.30)		1.80 (1.07–3.01)	
Visit shop before/after school ≥1 time week	NEV	447 (68.7)	1.00	< 0.001	1.00	< 0.001
	SEL	126 (19.4)	1.50 (0.98–2.28)		1.33 (0.93–1.84)	
	OFT	78 (12.0)	4.09 (2.48–6.73)		3.61 (2.17–6.01)	

^a^Adjusted for gender, self-efficacy, ethnicity, parental education, number of shops within walking distance from school

^b^Crude and adjusted odds ratios (cOR/aOR)

^c^NEV: adolescents never or rarely using the school canteen (*n* = 485); SEL: adolescents using the school canteen once a week (*n* = 142); OFT: adolescents using the school canteen ≥2 times a week (*n* = 93)

### Results of focus group and interview analyses

The data from the focus group interviews indicated that students were aware of issues related to food and health. A number of the relevant themes which emerged are outlined below.

### Student’s lunch habits

The majority of students confirmed that most foods consumed at school were brought from home. Some students, however, stated that the other option was to purchase foods from either the canteen or local shops:

*Interviewer: ….do you bring a packed lunch from home regularly?*

*Boy2: We usually tend to buy something from the canteen.*

*Girl5: It’s kind of both in a way.*

*Girl5: Yes. Ehm, it is usually both, there are many who have food with them also. Also you are free to buy something.*

*Boy1: Yes, that’s common…there are quite a few who tend to buy food at the canteen and, yes, the shop.*

One teacher suggested it was the presence of pocket money that determined the source of a student’s lunch:

*Teacher1: It is an incredibly large amount of money they have to buy canteen food with, especially in the 8th grade…so that means they do not have so much food with them from home, but buy it instead.*

### Types of foods purchased at school canteen, students’ impression of canteen

In response to the types of foods available for purchase at the canteen, student’s representing different schools reported similar food items. Overall, the students at all schools expressed a level of dissatisfaction with the healthiness of the food/drinks offered by the canteen:

*Interviewer: What is the most popular items people buy [at the canteen]?*

*Boy2: Mainly toasted sandwiches*

*Boy2: And wraps*

*Boy3: Eh, maybe a baguette with ham and cheese*

*Boy1: Whole-wheat bread with cheese and ham. Capsicum maybe.*

*Boy2: There are many different drinks one can buy, as well as yoghurt of various kinds. There is also a main thing available too, such as a baguette, pizza, or something similar.*

*Boy2: There are many who buy toasted sandwiches and wraps.*

*Interviewer: What can be done better in order to make other students or yourselves eat healthier from the school’s part?.*

*Girl3: They can begin to sell more fruit and such at the canteen.*

*Boy4: We could have healthier drink offers [from the canteen]…such as smoothies…*

*Girl2:…and switch chocolate milk with plain milk.*

*Boy3: [The canteen] should have healthier alternatives, not just unhealthy white-flour baguettes …with a little cheese, bit of ham and a little butter…..*

### Peer influence, perceived peer self-efficacy regarding healthy eating

There were questions designed to assess if students perceived other students as being more concerned with healthy eating. Those bringing food from home or considered ‘sporty’ were often perceived as eating healthy food, with the overall impression that those perceived as eating healthy tended to not purchase food at the canteen:

*Interviewer: …do you think there are some in your class then, that are more concerned with eating healthy than others?*

*Boy3: Yes, there are.*

*Interviewer: Who are they then?*

*Boy3: Those who ski.*

*Interviewer: How do you know that? Or, what is it that makes them stand out?*

*Boy2: They….don’t buy food at the canteen.*

*Boy4: They eat healthy food*

*Boy1: Those that eat relatively healthy food as a rule usually prepare food themselves.*

A number of school staff commented upon the influence some students’ lunch habits had upon others:

*Teacher6: …if there is one who begins to drop home brought food because it is boring, it become contagious over other’s behaviour I think, and then it isn’t cool to eat home packed lunches. They are at a very vulnerable age, and very affected by such things I believe.*

*Teacher2: …(food choices are affected by) what food they have at home, how much money they have in their pocket, and what their friends eat. I think it is these three things. And I think some….won’t bring out their home packed lunch because it is not cool enough.*

### Prices, timing, and permission for visiting shops

In many instances, it was reported that although leaving school grounds was not allowed during school hours in individual school policy, many students frequently did so in order to visit local food shops during breaks. There were reports of shop visits outside school hours as well (before/after school). Some students also discussed the cheaper prices at the shops, as compared to the school canteen, as being an incentive to purchase from shops.

*Girl2: We have some in the class that shoot off to the shops to buy some sort of fast food every day.*

*Interviewer: So you are allowed to leave the school in your free time to buy food?*

*Girl2: No, but after school or right before.*

*Girl4:......They go over [to the shops] when the lunch break starts, then you see them come back when everyone has to go outside then.*

*Boy4: Because then there are no teachers out......and then it is easy to take a trip to the shops and...*

*Boy1: Buy cheaper things. Because they sell at a high price here.*

The paradox between students visiting shops in school hours, although not allowed, was also pointed out by school staff:

*Teacher1: …no, it is not allowed (to go to the shops), but there are some that do it anyway.*

*Headmaster6: ...of course the schools must represent counterculture in some way….so our students go to the shops…and then they make use of the offers that are there…as long as they have money from home.*

*Teacher2: …and they prefer to go (to the shops) in a group at the same time, because it is social and fun.*

### Types of foods purchased in shops

When questioned about the types of items purchased at the shops, the majority were in consensus that unhealthy snacks such as sweets, baked goods, and soft-drinks were mainly purchased. No participant mentioned the purchase of healthy food from the shops.

*Interviewer: What do people mostly buy there then? You mentioned sweet buns..[Looks at Girl1]*

*Boy2: Both sweet buns and doughnuts.*

*Girl1: There are many that buy candy after school and such.*

*Boy4: There are always some who always have money and always buy candy and such. Just like one I know who bought 1 kg of gingerbread dough here after school one day and sat down and ate it.*

*Girl2: Mostly those….soft drinks*

*Girl1: Soft drinks*

*Boy1: Candy and ice-tea.*

*Boy2: People don’t buy food at the shop…most buy themselves candy.*

### Adherence of school administration to guidelines for school meals

When school staff were asked about the implementation of the latest guidelines from the Norwegian Directorate of Health, most pointed out that they already offered the suggested timespan suggested for lunch, whilst others had yet to read the document.

*Teacher1: We have heard there is something new that has come, but we have not spent a lot of time discussing it amongst ourselves.*

*Teacher2: No, no relationship with them (new guidelines). I'm not sure. We do not sell sodas and juice in the cafeteria, but they [students] have it from home.*

*Teacher3: Hehe, I don’t think I’ve seen them, no…(laughs).*

*Headmaster1: So, what we do is to make sure that they have a good place to eat and that they have peace….we offer supervision and they do have a long enough lunch break, is it 20 minutes they should have?*

*Headmaster2: I just have to be honest, I do not think we have come far with these.*

## Discussion

We found the NEV group were mainly female, having a high self-efficacy regarding the consumption of healthy foods, and with parents having an education over 12 years. By contrast, the OFT adolescents had a significantly higher proportion of males consuming salty snacks, baked sweets, and soft-drinks 3 or more times a week, as well as consuming breakfast less than 5 times a week when compared to the other groups, also when controlling for gender, parental education, self-efficacy, and use of shops (both during and before/after school).

When comparing the frequency of purchasing food and drink from local shops for these groups, we found the OFT group had a significantly higher proportion purchasing food/drink from shops near the school, both during the school break as well as before or after school, one or more times per week. Logistic regression analyses revealed the OFT group had nearly twice the odds for visiting shops during the school break, and significantly higher odds for visiting shops before/after school than the NEV group of adolescents.

Of the adolescents featured in this sample, females were revealed as more likely to never or rarely use the school canteen, a finding supported by previous research amongst adolescents [33, 34]. That females have been previously reported as having a greater self-efficacy related to healthy eating [35] may help to explain this result, although another study involving over 1200 students of comparable age found no significant difference in self-efficacy regarding gender [36]. As 67% of the sample stated that they never or rarely use the school canteen, this then begs the question of what form of lunch this group are consuming. Many of the interviews have mentioned the consumption of home packed lunches, and studies of school lunch habits amongst Norwegian adolescents have previously detailed the importance and predominance of the home packed lunch in Norwegian culture [37, 38], with over 60% of young Norwegians reporting a packed lunch for consumption at school, a proportion similar to the results we present here. This figure is also consistent with global reports examining school lunch eating practises [39].

Our results profile the OFT group as being mostly male, skipping breakfast, with a high frequency of shop visits during and on the way to/from school, and with a higher frequency of snacks, baked sweets, and soft-drinks, elements which have featured in previous studies regarding adolescent consumer behaviour [12, 40–43]. A clear association between adolescents skipping breakfast and subsequent purchases of foods from shops and fast food outlets, usually on the way to or from school [42, 44–46], in addition to other health-compromising behaviours [47] have been previously reported.

Although direct questions regarding pocket money were absent from our study, its role in the behaviour of this sample is evident from statements mentioning money use in the school administration interviews as well as alluded to in focus group interviews. Additionally, it stands to reason that adolescents using the school canteen often (i.e. the OFT group) would be equipped with money in order to make such purchases, as financial purchases are the norm in Norwegian secondary schools [48]. Research directed upon adolescents and pocket money has presented a number of findings that support our results regarding the OFT group, whereby access to spending money was associated with an increase of nutritionally poor food choices by adolescents, such as the increased consumption of fast-foods, soft-drinks, and unhealthy snacks off campus [40–43, 49–53]. These results may also be indicative of a gender imbalance in regards to pocket money provisions, where some studies report upon more males than females receiving pocket money [54, 55].

The mean age of this sample previously has been described as a stage in life of an emerging autonomy for young individuals, an autonomy which is exercised in terms of disposable income use and consumption of foods away from home [42, 56, 57]. This period of emerging autonomy may also manifest unhealthy eating behaviours as a strategy to forge identity amongst adolescents [58]. Frequent mention by students and staff in this study of themes relating to peer influence and defiance of school rules support the link between rebelliousness and unhealthy eating. Moreover, it has been reported previously that foods independently purchased by adolescents are often unhealthy, forbidden or frowned upon by parents, and express a defiant period of appearing ‘cool’ among peers, especially amongst males [37, 59–61], all of which support our findings here, particularly regarding gender, self-efficacy, and peer influence.

Value for money and dissatisfaction with the school canteen were frequently mentioned in the focus group interviews, and are elements that may be affecting choices made by the groups in this study. Statements concerning student dissatisfaction with canteen prices and/or the limited healthy options available have also appeared in previous research [35, 37, 38, 42]. That many of the school administrators interviewed seemed barely aware of the guidelines published by the Norwegian Directorate of Health is an alarming result, and likely adds some degree of weight upon student discontent with the school canteen. Although nearly all reports from the focus groups indicate the shops were used for unhealthy purchases, the possibility that shop purchases are a result of some adolescent’s need for healthier lunch alternatives cannot be dismissed completely.

The focus group interviews together with the quantitative data support the notion of healthy eaters avoiding the school canteen, opting instead for a home packed lunch. This view is further supported by previous reports that home prepared lunches help contribute to a healthy dietary pattern [39, 62, 63]. Furthermore, it has been reported that students consuming a lunch from home have significantly lower odds of consuming off-campus food during the school week [41], which further concurs with the results presented here.

By contrast, those often using the canteen – which, by all reports, could improve the healthiness of items offered – are using the off-campus shops often, purchasing mainly unhealthy snacks and drinks.

The strengths of the study include a large sample size with a high response rate at the school level, and moderate response rate at the parental level. Using a mixed method approach also provides a more comprehensive assessment of adolescent school lunch behaviours, allowing a fuller understanding of this and other adolescent food-behaviour settings by contrasting the adolescent’s own experiences with quantitative results. That the quantitative material, based on cross-sectional data, precludes any opportunity for causal inference to be made may be one of the prime weaknesses of this study. Quantitative data regarding adherence to national policy regarding school canteens, pocket money and what items it was spent upon, as well as data regarding the content and frequency of home packed lunch consumption, were also lacking from the study, where inclusion of these elements in the various analyses would have considerably strengthened the quality of results. Furthermore, reliance upon self-reported data may have led to issues regarding validity and reliability, particularly with a sample of young adolescents.

## Conclusion

We found the majority of adolescents (67.4%) in this sample rarely or never used the school canteen. Those adolescents using the school canteen two or more times a week were also the group most likely to be purchasing food/drink from a shop near the school, either during school breaks or before/after school. This group also tended to skip breakfast and consume snacks and soft-drinks more frequently compared to the adolescents who rarely or never used the school canteen. These findings highlight a lack of satisfaction of items available for consumption at the school canteen, with adolescents intending to use the school canteen preferring instead the shops for foods that are cheaper and more desirable. Future strategies aimed at improving school food environments need to address the elements of value for money and appealing healthy food availability in the school canteen, as well as elements such as peer perception and self-identity attained from adolescent food choices, especially in contrast to the competitiveness of foods offered by nearby food outlets.

## Additional files

Additional file 1: Appendix 1. ESSENS questionnaire relating to food behaviours ESSENS Study. (DOCX 33 kb)

Additional file 2: Appendix 2. Interview guide for focus group interviews. (DOCX 13 kb)

Additional file 3: Appendix 3. Interview guide for headmasters and teachers. (DOCX 14 kb)

## Abbreviations

aOR: Adjusted odds ratio
CI: Confidence interval
cOR: Crude odds ratio
ESSENS: Environmental determinantS of dietary behaviorS among adolescENtS study
NEV: Adolescents never or rarely using the school canteen
NOK: Norwegian kroner
OFT: Adolescents using the school canteen two or more times a week
SEL: Adolescents using the school canteen once a week
