# Supplementary material for: Consumption habits of school canteen and non-canteen users among Norwegian young adolescents: a mixed method analysis
Source: BMC Pediatr. 2018 Oct 16;18:328. doi: 10.1186/s12887-018-1299-0 (PMC6192152; doi:10.1186/s12887-018-1299-0)
Supplement: Supplementary file 1 — Appendix 1. ESSENS questionnaire relating to food behaviours ESSENS Study. (DOCX 33 kb) [file 12887_2018_1299_MOESM1_ESM.docx]

**Appendix 1: ESSENS questionnaire relating to food behaviours**

**ESSENS Study**

**DIET**

**How often do you usually eat FRESH FRUIT?**

Never/rarely

Less than 1 time a week

1-2 times per week

3-4 times per week

5-6 times per week

1 time per day

2 times per day

3 or more times per day

**How often do you usually eat RAW VEGETABLES (e.g. carrot, tomato, salad)?**

Never/rarely

Less than 1 time a week

1-2 times per week

3-4 times per week

5-6 times per week

1 time per day

2 times per day

3 or more times per day

**How often do you usually eat WARM VEGETABLES (NOT potatoes)?**

Never/rarely

Less than 1 time a week

1-2 times per week

3-4 times per week

5-6 times per week

1 time per day

2 times per day

3 or more times per day

**How often do you usually eat CHOCOLATE/SWEETS?**

Never/rarely

Less than 1 time a week

1-2 times per week

3-4 times per week

5-6 times per week

1 time per day

2 or more times per day

**How often do you usually eat SALTY SNACKS? (e.g. potato chips, popcorn and similar)**

Never/rarely

Less than 1 time a week

1-2 times per week

3-4 times per week

5-6 times per week

1 time per day

2 or more times per day

**How often do you usually eat SWEET BISCUITS, BUNS, MUFFINS or similar?**

Never/rarely

Less than 1 time a week

1-2 times per week

3-4 times per week

5-6 times per week

1 time per day

2 or more times per day

**ON WEEKDAYS (Monday to Friday), how often do you normally drink the following types of beverages, and how much per day?**

**Soft drinks with sugar (e.g. Cola, Solo)**

Never/rarely

1 day per week

2 days per week

3 days per week

4 days per week

Every day

**When you drink soft drinks with sugar during the week, how MUCH do you consume?**

(1/2 liter = 3 glasses)

1 glass

2 glasses

3 glasses

4 glasses or more

**ON WEEKENDS:**

**How much of the following drinks do you consume? (1/2 liter = 3 glasses). Combine amounts you drink on Saturday and Sunday, and place a mark for every type of drink.**

**Soft drinks with sugar (e.g. Cola, Solo)**

Never/rarely

1 glass

2 glasses

3 glasses

4 glasses

5 glasses

6 glasses

**How much do you agree or disagree with the following statements**

| Place only one mark  **At my home..** | **Completely disagree** | **Disagree slightly** | **Neither agree nor disagree** | **Agree slightly** | **Completely agree** |
| --- | --- | --- | --- | --- | --- |
| ..we USUALLY have vegetables for dinner every day | ⬜ | ⬜ | ⬜ | ⬜ | ⬜ |
| .. we vary the TYPE of vegetables served for dinner during the week | ⬜ | ⬜ | ⬜ | ⬜ | ⬜ |
| .. we vary the PREPARATION of vegetables (raw, warm, etc) served for dinner during the week | ⬜ | ⬜ | ⬜ | ⬜ | ⬜ |
| .. there are USUALLY vegetables I like available | ⬜ | ⬜ | ⬜ | ⬜ | ⬜ |
| .. I can eat vegetables when I want | ⬜ | ⬜ | ⬜ | ⬜ | ⬜ |
| .. I can eat as much vegetables I want | ⬜ | ⬜ | ⬜ | ⬜ | ⬜ |
| .. my parents prepare vegetables for me to eat between meals | ⬜ | ⬜ | ⬜ | ⬜ | ⬜ |

**How much do you agree or disagree with the following statements**

| Place only one mark  **At my home..** | **Completely disagree** | **Disagree slightly** | **Neither agree nor disagree** | **Agree slightly** | **Completely agree** |
| --- | --- | --- | --- | --- | --- |
| .. there is USUALLY fruit I like available | ⬜ | ⬜ | ⬜ | ⬜ | ⬜ |
| .. we vary the TYPE of fruit available during the week | ⬜ | ⬜ | ⬜ | ⬜ | ⬜ |
| .. my parents prepare fruit for me to eat between meals | ⬜ | ⬜ | ⬜ | ⬜ | ⬜ |
| .. I can eat fruit when I want | ⬜ | ⬜ | ⬜ | ⬜ | ⬜ |
| .. I can eat as much fruit I want | ⬜ | ⬜ | ⬜ | ⬜ | ⬜ |
| ..my mother eats fruit every day | ⬜ | ⬜ | ⬜ | ⬜ | ⬜ |
| ..my mother eats vegetables every day | ⬜ | ⬜ | ⬜ | ⬜ | ⬜ |
| ..my father eats fruit every day | ⬜ | ⬜ | ⬜ | ⬜ | ⬜ |
| ..my father eats vegetables every day | ⬜ | ⬜ | ⬜ | ⬜ | ⬜ |

**How much do you agree or disagree with the following statements**

| Place only one mark  **At my home..** | **Completely disagree** | **Disagree slightly** | **Neither agree nor disagree** | **Agree slightly** | **Completely agree** |
| --- | --- | --- | --- | --- | --- |
| ..we have rules about when I can drink soft drinks with sugar | ⬜ | ⬜ | ⬜ | ⬜ | ⬜ |
| ..we have rules about how much soft drinks with sugar I can drink | ⬜ | ⬜ | ⬜ | ⬜ | ⬜ |
| .. I can drink soft drinks with sugar when I want | ⬜ | ⬜ | ⬜ | ⬜ | ⬜ |
| .. I can drink as much soft drinks with sugar as I want | ⬜ | ⬜ | ⬜ | ⬜ | ⬜ |
| .. there is USUALLY soft drinks with sugar available | ⬜ | ⬜ | ⬜ | ⬜ | ⬜ |
| .. there is USUALLY soft drinks with sugar available at dinner during the WEEKDAY | ⬜ | ⬜ | ⬜ | ⬜ | ⬜ |
| .. there is USUALLY soft drinks with sugar available at dinner during the WEEKEND | ⬜ | ⬜ | ⬜ | ⬜ | ⬜ |

**How often do your parents/guardians drink soft drinks or cordial?**

Always

Often

Sometimes

Rarely

Never

PARENST ADMINISTRATION OF FOOD AND DRINK

These statements focus on foods your mother/father discuss regarding amounts of sugars, sweets or snacks consumed. The scale runs from 1 to 5. Answer option 1 equates to «never» and answer option 5 equates to «always». Mark the option (1-5) that you think best represents your mother and father

| **1** | **2** | **3** | **4** | | **5** |
| --- | --- | --- | --- | --- | --- |
| **Never** |  |  |  | | **Always** |
| My MOTHER places clear restrictions regarding how much sugar soft drinks (cola, cordial) I can drink | | | | 1 2 3 4 5 | |
| My FATHER places clear restrictions regarding how much sugar soft drinks (cola, cordial) I can drink | | | | 1 2 3 4 5 | |
| My MOTHER places clear restrictions regarding how much sweet items (ice cream, cookies, cakes) I can eat | | | | 1 2 3 4 5 | |
| My FATHER places clear restrictions regarding how much sweet items (ice cream, cookies, cakes) I can eat | | | | 1 2 3 4 5 | |
| My MOTHER places clear restrictions regarding how much fatty snacks (potato chips, salty peanuts etc) I can eat | | | | 1 2 3 4 5 | |
| My FATHER places clear restrictions regarding how much fatty snacks (potato chips, salty peanuts etc) I can eat | | | | 1 2 3 4 5 | |

**How much do you agree or disagree with the following statements**

| Place only one mark  **At my home..** | **Completely disagree** | **Disagree slightly** | **Neither agree nor disagree** | **Agree slightly** | **Completely agree** |
| --- | --- | --- | --- | --- | --- |
| .. there are usually sweet or fatty snacks available | ⬜ | ⬜ | ⬜ | ⬜ | ⬜ |
| .. there are usually sweet or fatty snacks served as dessert or snacks on WEEKDAYS | ⬜ | ⬜ | ⬜ | ⬜ | ⬜ |
| .. there are usually sweet or fatty snacks served as dessert or snacks on WEEKENDS | ⬜ | ⬜ | ⬜ | ⬜ | ⬜ |
| .. when we have sweet or fatty snacks available, I can eat some when I want | ⬜ | ⬜ | ⬜ | ⬜ | ⬜ |

**How often do your parents/guardians eat sweet or fatty snacks?**

Always

Often

Sometimes

Rarely

Never

**How much do you agree or disagree with the following statements**

| Place only one mark  **When I am free to choose what I can eat, I…** | **Completely disagree** | **Disagree slightly** | **Neither agree nor disagree** | **Agree slightly** | **Completely agree** |
| --- | --- | --- | --- | --- | --- |
| .. think it is difficult to choose food with a low fat content (e.g. fruit instead of potato chips) | ⬜ | ⬜ | ⬜ | ⬜ | ⬜ |
| .. think it is easy to choose a healthy mid-meal snack (e.g. fruit or lite yoghurt) | ⬜ | ⬜ | ⬜ | ⬜ | ⬜ |
| .. think I know how to choose or make healthy food | ⬜ | ⬜ | ⬜ | ⬜ | ⬜ |
| .. think it is difficult to choose healthy meals/snacks when I am together with friends | ⬜ | ⬜ | ⬜ | ⬜ | ⬜ |
| .. think it is easy to eat at least 5 portions of fruit and vegetables every day | ⬜ | ⬜ | ⬜ | ⬜ | ⬜ |
| .. think it is easy to eat acceptable meal portions (not eat until I am feeling full) | ⬜ | ⬜ | ⬜ | ⬜ | ⬜ |

**In a normal school week from Monday to Friday, how many days do you eat breakfast?**

I never eat breakfast on school days

1 day

2 days

3 days

4 days

5 days

**How often do you eat breakfast on weekends? (Saturday and Sunday)**

I never eat breakfast on weekends

I usually eat breakfast Saturday OR Sunday

I usually eat breakfast on Saturday AND Sunday

**How often do your parents/guardians eat breakfast**

Always

Often

Sometimes

Rarely

Never

**How often do you eat breakfast with your parents/guardians?**

Always

Less than 1 day in the week

1 day per week

2-4 days per week

5-6 days per week

Every day

**Is there usually breakfast foods (bread, breakfast cereal, milk) at home?**

Always

Often

Sometimes

Rarely

Never

**My parents/guardians have rules regarding my breakfast consumption**

Completely disagree

Disagree slightly

Neither agree nor disagree

Agree slightly

Completely agree

**Is there a shop (supermarket, kiosk, petrol station) on your way to/from school?**

Yes, one

Yes, two

Yes, three or more

No

**How often do you usually buy food/drink in these shops in your school breaks?**

There are no shops close to my school

Never

1 day per week

2 days per week

3 days per week

4 days per week

Every day

**How often do you usually buy food/drink from shops around your school or neighbourhood on your way to/from school?**

Never

1 day per week

2 days per week

3 days per week

4 days per week

Every day

**How often do you buy food from the school cantine?**

We have no school canteen

Never

1 day per week

2 days per week

3 days per week

4 days per week

Every day

**In what degree are you in agreement with the following statements regarding shops near your school or neighbourhood where you purchase food and drink?**

| Place only one mark | **Completely disagree** | **Disagree slightly** | **Neither agree nor disagree** | **Agree slightly** | **Completely agree** |
| --- | --- | --- | --- | --- | --- |
| .. there are a lot of fresh and varied fruits and vegetables I like available | ⬜ | ⬜ | ⬜ | ⬜ | ⬜ |
| .. there are a lot of sweets and fatty snacks (e.g. chocolate, biscuits, muffins, potato chips) I like available | ⬜ | ⬜ | ⬜ | ⬜ | ⬜ |
| .. there are a lot of fruit and vegetables available in a form easy to consume | ⬜ | ⬜ | ⬜ | ⬜ | ⬜ |
| .. it is cheaper to buy soft drinks or snacks (e.g. biscuits or potato chips) then it is to buy fruit and vegetables | ⬜ | ⬜ | ⬜ | ⬜ | ⬜ |
| .. it is easier to obtain soft drinks or snacks in the shop then it is to obtain fruit or vegetables | ⬜ | ⬜ | ⬜ | ⬜ | ⬜ |
