# Supplementary material for: Consumption habits of school canteen and non-canteen users among Norwegian young adolescents: a mixed method analysis
Source: BMC Pediatr. 2018 Oct 16;18:328. doi: 10.1186/s12887-018-1299-0 (PMC6192152; doi:10.1186/s12887-018-1299-0)
Supplement: Supplementary file 2 — Appendix 2. Interview guide for focus group interviews. (DOCX 13 kb) [file 12887_2018_1299_MOESM2_ESM.docx]

| **Individuals** | Eating habits  o Can you tell me what is most often here at school?  o What do you associate with healthy food?  o What do you consider ‘unhealthy’ foods?  o Is it important for you to have a healthy diet? Is there anybody that is especially focussed on having a healthy diet? Who are they? |
| --- | --- |
| **Sosial environment** | o How would you describe a regular lunch break at school?  o Do you experience that there is a focus on healthy food and physical activity in others your class? Among students, teachers, other employees?  o Do you experience the food you eat is influenced by people around you? (friends, family, teachers)  o What responsibility do you think the school should have in order to contribute to healthy eating habits amongst its’ students?  o Do you experience the school taking responsibility when it comes to promoting healthy eating habits and physical activity at school? In what way?  o Who else do you think should take responsibility for students eating healthy food at school?  o Have you experienced any obstacles to eating healthy food at school? What are these?  o Are there any situations you think it is easier to eat healthy food at school?  o Is it important for you to be in activity during the school day? Can you tell me something about why? |
| **Organisation** | What do you think about the free school fruit programme being removed?  o Do you get offers regarding fruit and/or milk at school?  o Do you think there is something teachers at school could do to encourage healthier food choices among students in your class?  o What do you think about the canteen selling foods like soft drinks, cakes and chocolate?  o If you do not bring a packed lunch from home, do you choose to buy food from the school's canteen or from shops in the area? What do you eat there and why?  o Is there something that the school could do differently to encourage more physical activity among students in your class? |
| **Environment** | What do you think about students being allowed to leave school during lunch break (s) to visit the shops/kiosks in the area? Do you think it's a good idea? Why?  o If you leave school to go to the store, what is the reason for that?  o Can you tell me about the surrounding area around their school?  Do you experience it's safe to walk or ride to/from school?  o Do you experience that it is encouraged to be active in the school area and in the immediate area? In what way? |

**Appendix 2: Interview guide for focus group interviews**
