# Supplementary material for: Consumption habits of school canteen and non-canteen users among Norwegian young adolescents: a mixed method analysis
Source: BMC Pediatr. 2018 Oct 16;18:328. doi: 10.1186/s12887-018-1299-0 (PMC6192152; doi:10.1186/s12887-018-1299-0)
Supplement: Supplementary file 3 — Appendix 3. Interview guide for headmasters and teachers. (DOCX 14 kb) [file 12887_2018_1299_MOESM3_ESM.docx]

**Appendix 3: Interview guide for headmasters and teachers**

**Organization of food and meals at school**

1. Can you tell me how the lunch break is organized here at school? (Time of day, subsidy plans for fruit and vegetables/milk? Supervision, where do they eat?)

2. How is the food break between teachers and staff at school experienced?

3. Can you tell me a little about how the canteen is organized? Procedure of buying food there?

4. What is your opinion of the foods offered?

5. Dialogue between management and the cafeteria? How much does the school affect the food supply in the cafeteria? Can you come up with examples?

6. Are students allowed to leave the school area in their free time? What do you think about that? Why did you choose to introduce that rule?

7. What do you think about students buying food and drink in shops/kiosks? Are they returning to school with foods? Do you think that affects the other students who do not have the opportunity to buy something?

8. Has the school had any contact with the local stores? Can you tell me something about that?

**Attitudes towards food / school responsibility**

9. Can you tell me about which factors you think affect the students' eating habits at school?

(Parents, teachers, rules/bans at school, canteen, group press?)

10. Are the eating habits of the students a topic discussed among the teachers? Do you remember if this topic was ever discussed? In what connection? Among which teachers?

11. Does the management at school have any concerns or thoughts regarding the food and health of the students? Eating Disorders? Overweight among the children at school? Are there any groups that are particularly susceptible? What are your thoughts about it? Is something being done in this area?

12. Do you think the school is in charge of young people's diet? In what areas?

13. What measures do you think can improve the students diet at school? Can you come up with examples?

14. Have soft drinks or other energy drinks been a theme at school? In what connection? What are your thoughts about it?

15. Does the school have any resources that can be used to encourage healthy eating habits? What will be needed?

**Guidelines for food and meals at school**

This autumn a new guideline for food and meals in school was launched (previous version from 2003). In the new version, they have emphasized how the school should arrange food and meals at school, the structure around the mealtimes and detailed advice on how this can be achieved in practice.

Theme: The foods offered in the canteen (wholegrain cereal products, fish spreads, fruit and vegetables, water, ban on soda, energy drinks), the structure around the meal (hand wash, enough time to eat, adult supervision), regular meals that promote food pleasure, social engagement, well-being and health (areas for dining, offering food to those who do not have/have money).

16. How does school relate themselves to the national guidelines for the school meal?

17. Are there any areas that you think are challenging to provide at school? Planned to implement?

18. What do you think about the guidelines - useful tools?

19. Has the school introduced its own rules for food and drink? Can you tell me something about them? Why did you choose to do that? How long have they been in used?

20. Do you think it seems that most teachers/employees follow these rules?

21. Is food and nutrition a priority area beyond that which is expected at school? Can you explain a little more about it?

**Food and health classes**

22. Can you tell me a little about the organization of food and health classes?

23. Who can teach in food and health classes? Teacher? Substitutes? Assistants?

24. What is emphasised on food and health education classes?

(Food culture, dining, practice in the kitchen, healthy food, lifestyle, experimentation in the kitchen?)

25. Can food and health classes contribute to value creation amongst young people?

26. Do you think learning outcomes from food and health classes can affect the eating habits of students outside school? In what situations?
